# Local uniqueness solution of illuminated solar cell intrinsic electrical parameters

**DOI:** 10.1186/2193-1801-3-152

**Published:** 2014-03-20

**Authors:** Abdennaceur Jarray, Mahdi Abdelkrim, Mohamed Bouchiba, Abderrahman Boukricha

**Affiliations:** Faculté des Sciences de Tunis, Unité de recherche: Optimisation Appliquée, Campus Universitaire, 1060 Tunis, Tunisie; Instiut National des Sciences Appliquées et de Technologie, Centre urbain Nord de Tunis, BP 676, 1080 Tunis, Tunisie

**Keywords:** Solar cell model, Electrical parameters, Electrical characterization, Lambert function, Shokley’s equation, Numerical modeling

## Abstract

Starting from an electrical dissipative illuminated one-diode solar cell with a given model data at room temperature (*I*_*sc*_, *V*_*oc*_, *R*_*s0*_, *R*_*sh0*_, *I*_*max*_); we present under physical considerations a specific mathematical method (using the Lambert function) for unique determination of the intrinsic electrical parameters (*n*, *I*_*s*_, *I*_*ph*_, *R*_*s*_, *R*_*sh*_). This work proves that for a given arbitrary fixed shunt resistance *R*_*sh*_, the saturation current *I*_*S*_ and the ideality factor *n* are uniquely determined as a function of the photocurrent *I*_*ph*_, and the series resistance *R*_*s*_. The correspondence under the cited physical considerations: *R*_*s*_ does not exceed ]0, 20[*Ω* and *n* is between ]0, 3[ and *I*_*ph*_ and *I*_*s*_ are arbitrary positive

, is biunivocal. This study concludes that for both considered solar cells, the five intrinsic electrical parameters that were determined numerically are unique.

## Introduction

Although the electrical dissipative one diode model has a potential of improvement in the efficiency and the stability of the solar cell structure under illumination, to our knowledge the uniqueness and the authenticity of the extracted intrinsic electrical parameters associated to the model have not been studied previously.

In this work we attempt to develop this concept and prove the uniqueness of the determination of these parameters.

The one-diode model gives sufficient efficiency for earthly applications (Charles 1984). A precise numerical method using this model was presented in the early 1980s by Charles *et al.* (1981; 1985).

.The use of the Lambert W-Function proposed by Corless *et al* (1996) allowed demonstrating explicitly the Shokley’s modified eq. (1) which is related to the equivalent electrical circuit model as shown in Figure 1.1

**Figure 1:**
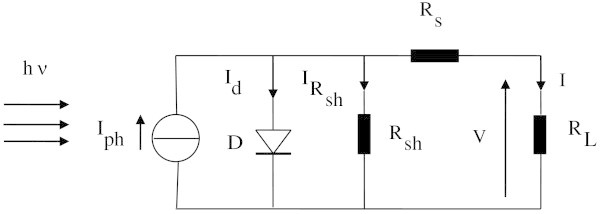
**Solar cell one-diode equivalent circuit model, under specified illumination and temperature.**

Where *I*_*ph*_ is the photocurrent, *n* is the diode ideality factor of the junction, *I*_*s*_is the reverse saturation current, *R*_*s*_is the series resistance and *R*_*sh*_ is the shunt resistance.

Each of these parameters is connected to the suited internal physical mechanism acting within the solar cell. Their knowledge is therefore important.

Several methods were proposed to determine the intrinsic electrical parameters: *I*_*ph*_*; n; I*_*s*_*; R*_*s*_*; R*_*sh*_ presented in eq. (1) of the solar cell. In particular, Jain and Kapoor (2005) established a practical method to determine the diode ideality factor of the solar cell.

Ortiz-Conde *et al.* (2006) have used a co-content function to determine these parameters. Jain *et al.* (2006) determine these parameters on solar panels. Chegaar *et al.* (2006) have used four comparative methods to determine these parameters.

More recently, Kim and Choi (2010) have used another method to determine the intrinsic parameters of the cell by making a remarkable initialization of the ideality factor *n* and the saturation current *I*_*s*_ (Kim & Choi 2010).

## Theoretical study: problem formulation

To determine the solar cell intrinsic electrical parameters (*n, I*_*s*_*, I*_*ph*_*, R*_*s*_*, R*_*sh*_), we put together a system of five equations (Lemma 2), and, solved by two different numerical methods. The Lambert W-function is the reverse of the function F defined from *C*_*+*_ in *C* by *F* (*W*)  = *W e*^*W*^ for every W in *C*_*+*_.

**Lemma 1**: The Lambert W-function was derived from eq. (1) by expressing the current *I* in function of the voltage *V* and vice-versa, as follows234

**Figure 2:**
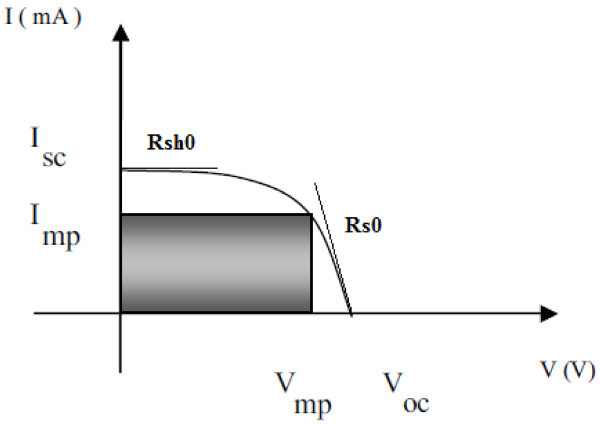
**I (V) characteristics of a solar cell under illumination in generator convention.**

We consider the following I (V) solar cell characteristics under illumination in generator convention as presented in Figure 2.

Where *I*_*sc*_ and *V*_*oc*_ represent the short-circuit current and the open-circuit voltage respectively, *R*_*sh0*_ is the slope of the I-V curve at the (0, *I*_*sc*_) point, *R*_*s0*_ is the slope of the I-V curve at the (*V*_*oc*_, 0) point and *I*_*max*_ is the maximum power current, and *I*_*ph*_*, I*_*s*_*, n, R*_*s*_, and *R*_*sh*_ are the intrinsic electrical parameters that should be determined.

In order to simplify the problem formulation, we adopt the following abbreviations

From eq. (2) and at the point (0, Isc) we obtained5

Idem from eq. (3) and at the point (V_oc_, 0) we obtained6

The slope at the point (V_oc_, 0) of the eq. (2) we obtained7

The slope at the point (0, Isc) of the eq. (2) gives8

For differentiating eq. (4) and at the point (I = I_max_) stems9

**Lemma 2**: We have the following system10

**Proof**: For *I = I*_*sc*_ and *V = 0*, eq. (2) implies that *f*_*1*_(*X*, *Y*) = *0* and for *V = V*_*oc*_ and *I = 0* eq. (3) implies that *f*_*2*_(*X*, *Y*) = *0*.

The differential resistances: *R*_*s0*_ and *R*_*sh0*_ lead to the following two equations: *f*_*3*_(*X*, *Y*) = *0*  and *f*_*4*_(*X*, *Y*) = *0*.

From eq. (4), maximal power obtained by:  implies that *f*_5_(*X*, *Y*) = 0.

In order to solve the system presented in Lemma 2 (eq. 10) and determine the intrinsic electrical parameters, a set of experimental measurements (data) were used (Table 1).

**Table 1:** **SAT and Cu**_**2**_ **S-CdS cells experimental data**

Experimental data	SAT cell (E = 1 S)	Cu_2_S-CdS cell (E = 1 S)
*V*_*oc*_ *(V)*	0.536	0.469
*R*_*s0*_ *(Ω)*	0.45	6.857
*I*_*sc*_ *(A)*	0.1025	0.04075
*I*_*max*_ *(A)*	0.0925	0.025
*R*_*sh0*_ *(Ω)*	1000	41.905
*V*_*T*_ *(V)*	0.025875	0.023527

These measurements were collected from two different solar cells under AM1 illumination (E = 1 S = 100 mW/cm^2^) at room temperature.

Our study concerns p-n junctions at both homo- and hetero-junctions: For the homo-junction, a 4 cm^2^ blue type monocrystalline silicon cell produced by SAT (1980) was used. For the hetero-junction we have used a frontwall Cu_2_S-CdS cell produced by a wet (Cleveite) process with significant losses of 4.28 cm^2^ square area. Two different numerical methods were applied in order to prove their authenticity.

## Numerical approach of the intrinsic parameters

### Newton’s method

The following function was considered

Let J_F_ denote the Jacobian matrix defined by

So, Newton’s method can be formulated as follows: For  as an initial condition and for all *k* = 0, 1 … until convergence; we have to resolve the unknown variable *Y*^*k*^ using the following system of equations: *J*_*F*_ (Y^k^) *δ* Y^k^ = ‒ F (Y^k^), where: *Y*^*k* + 1^ = Y^k^ + *δ* Y^k^ and: .

In order to apply the Newton’s method to this system an iterative program was developed in a MAPLE environment Monagan *et al.* (2003) using an accuracy of 20-digits.

It depends on the choice of the initial data *Y*^*0*^ by making sure that *J*_*F*_ (*Y*^k^) ≠ 0 and by continuing the iteration process until a quadratic convergence is reached.

At each increment, the program performs a test between two successive iterations by assessing the Euclidean norm of their difference. The program was designed to stop the calculation when the test reaches a value smaller than the pre-set tolerance value.

### Hooke-Jeeves’s method

The Hooke-Jeeves method is based on numerical calculation of the minimum of a function *G* without the use of gradient. This method is widely used in applications with convex *G*.

This method was used in this study to find the zero of function *G* (eq. 11) by minimizing in *X* and *Y* such that11

We recall that *G (X, Y)* = 0 is equivalent to the system presented in eq. (9) which leads to the determination of the intrinsic electrical parameters.

This method has the advantage of being easily programmed except the need to calculate gradient *G*.

## Existence and uniqueness of the solution

To determine the existence and the uniqueness of the system presented in lemma 2 (eq. 9), we use the following implicit functions theorem where: *H* represents a continuously differentiable real-valued functions defined on a domain *D* in *IR*^*2*^*x IR*^*2*^ into *IR*^*2*^:

By using the following notations: *A* = (*I*_*ph*_, *R*_*s*_); *B* = (*n*, *I*_*s*_)

Let  be the following Jacobian matrix:

Let: (*A*^0^,  *B*^0^) be a point in *D* such that *H* (*A*^0^,  *B*^0^) = 0, and  is invertible i.e. .

The last step is to determine the neighborhood *U* × *V* where the following determinant of the Jacobian matrix will remain

**Figure 3:**
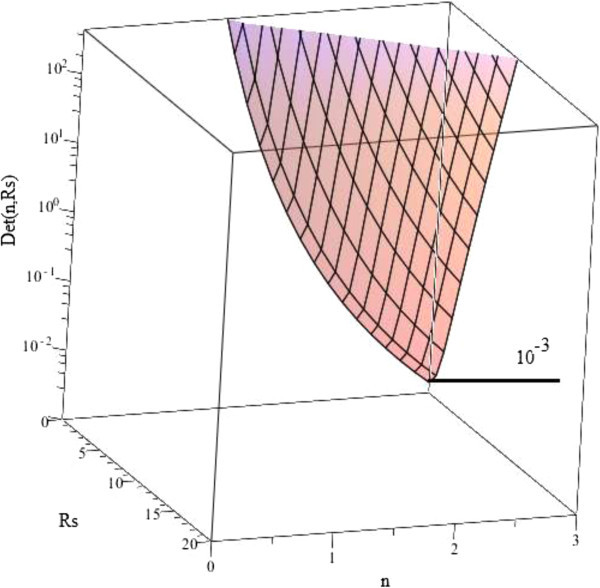
**R**_**s**_ **and n dependences of Det (R**_**s**_ **, n).**

This determinant does not depend on *I*_*ph*_ and is linear with *I*_*s*_. The *R*_*s*_ and *n* dependences of the determinant are illustrated in the following figure (Figure 3).

The minimum of the determinant in ] 0, 20[×]0, 3 [ is 10^-3^. Consequently the investigated neighborhood *U* × *V* is 

The implicit functions theorem gives the existence of a unique function *B* = *ϕ* (A) defined in *U* into *V* of class *C*^*1*^ and for any (*A*, *B*) ∈ *U* × *V*, *H* (*A*, *ϕ* (*A*)) = 0. As a result the *φ* Jacobian matrix is given by the formula:  and consequently, we prove for a given arbitrary fixed shunt resistance *R*_*sh*_, that the saturation current *I*_*s*_ and the ideality factor *n* are uniquely determined in function of the photocurrent *I*_*ph*_, and the series resistance *R*_*s*_.

## Experimental and theoretical results, discussion of related authenticity

Tables 2 and 3 list the intrinsic electrical parameters values of the two cells determined by Newton’s method and Hooke-Jeeves’s.

**Table 2:** **SAT solar cell’s intrinsic electrical parameters (E = 1 S)**

Intrinsic parameters	Newton’s method	Hooke-Jeeve’s method
*I*_*ph*_ *(A)*	0.102502	0.102002
*I*_*s*_ *(A)*	5.987171985 × 10^-7^	5.97501 × 10^-7^
*n*	1.709464	1.721481
*R*_*s*_ *(Ω)*	0.016437	0.016437
*R*_*sh*_ *(Ω)*	1014.244754	1000.412260

**Table 3:** **Frontwall Cu**_**2**_ **S-CdS solar cell’s intrinsic electrical parameters (E = 1 S)**

Intrinsic parameters	Newton’s method	Hooke-Jeeve’s method
*I*_*ph*_ *(A)*	0.045528	0.045487
*I*_*s*_ *(A)*	8.2455 × 10^-6^	8.0 × 10^-6^
*n*	2.183476	2.176611
*R*_*s*_ *(Ω)*	5.355408	5.298477188
*R*_*sh*_ *(Ω)*	49.838828	49.175974

To prove the authenticity of the model, we should calculate the current *I* listed as *I*_*th*_ by the use of the obtained intrinsic parameters at different points of the I-V curves. These points are compared with the corresponding experimental current values listed as *I*_*exp*_. The accuracy is evaluated by the parameter *D (%).* The values of the called accuracy *D* (%*)* corresponding to the percentage deviation between experimental and theoretical results are also listed in Tables 4, 5, 6 and 7 and does not exceed 0.2%.

**Table 4:** **SAT solar cell’s calculated I-V values by Hooke’s method**

***V (V)***	***I***_***exp***_ ***(A)***	***I***_***Hooke***_ ***(A)***	***D (%)***
0.000000	0.102500	0.102501	0.000009
0.100000	0.102500	0.102396	0.001015
0.150000	0.102500	0.102333	0.001631
0.200000	0.102500	0.102245	0.002494
0.250000	0.102500	0.102079	0.004124
0.300000	0.101500	0.101669	0.001665
0.325000	0.101200	0.101241	0.000405
0.350000	0.100500	0.100510	0.000099
0.375000	0.099500	0.099246	0.002559
0.400000	0.09770	0.097048	0.006718
0.425000	0.09450	0.093216	0.013774
0.450000	0.08900	0.086533	0.028509
0.475000	0.07780	0.074895	0.038787
0.500000	0.05750	0.054718	0.050842
0.536000	0.00000	0.000000	0.000000

**Table 5:** **SAT solar cell’s calculated I-V values by Newton’s method**

***V (V)***	***I***_***exp***_ ***(A)***	***I***_***Newton***_ ***(A)***	***D (%)***
0.000000	0.102500	0.102500	0.000000
0.100000	0.102500	0.102397	0.001005
0.150000	0.102500	0.102337	0.001592
0.200000	0.102500	0.102255	0.002395
0.250000	0.102500	0.102104	0.003878
0.300000	0.101500	0.101739	0.002354
0.325000	0.101200	0.101359	0.001571
0.350000	0.100500	0.100708	0.002069
0.375000	0.099500	0.099580	0.000804
0.400000	0.09770	0.097614	0.000881
0.425000	0.09450	0.094173	0.003472
0.450000	0.08900	0.088149	0.009654
0.475000	0.07780	0.077613	0.002409
0.500000	0.05750	0.059257	0.030556
0.536000	0.00000	0.000000	0.000000

**Table 6:** **Frontwall Cu**_**2**_ **S-CdS solar cell’s calculated I-V values by Hooke’s method**

***V(V)***	***I***_***exp***_ ***(A)***	***I***_***Hooke***_ ***(A)***	***D(%)***
0.000000	0.0408000	0.041010	0.0051470
0.050000	0.0393	0.039746	0.011348
0.100000	0.0373	0.038120	0.021983
0.200000	0.0315	0.032689	0.037746
0.250000	0.0273	0.028445	0.041941
0.275000	0.0250	0.025929	0.037160
0.300000	0.0225	0.023173	0.029911
0.325000	0.0196	0.020201	0.030663
0.350000	0.0165	0.017038	0.032606
0.375000	0.0132	0.013705	0.038257
0.400000	0.0099	0.010225	0.032828
0.450000	0.0025	0.002895	0.158000
0.469000	0.000000	0.000000	0.000000

**Table 7:** **Frontwall Cu**_**2**_ **S-CdS solar cell’s calculated I-V values by Newton’s method**

***V(V)***	***I***_***exp***_ ***(A)***	***I***_***Newton***_ ***(A)***	***D(%)***
0.000000	0.0408000	0.040750	0.001226
0.050000	0.0393	0.039420	0.003053
0.100000	0.0373	0.037667	0.009839
0.200000	0.0315	0.031870	0.011746
0.250000	0.0273	0.027522	0.008131
0.275000	0.0250	0.025000	0.000000
0.300000	0.0225	0.022273	0.010191
0.325000	0.0196	0.019362	0.012292
0.350000	0.0165	0.016290	0.012891
0.375000	0.0132	0.013075	0.009560
0.400000	0.0099	0.009737	0.016740
0.450000	0.0025	0.002748	0.099200
0.469000	0.000000	0.000000	0.000000

**Figure 4:**
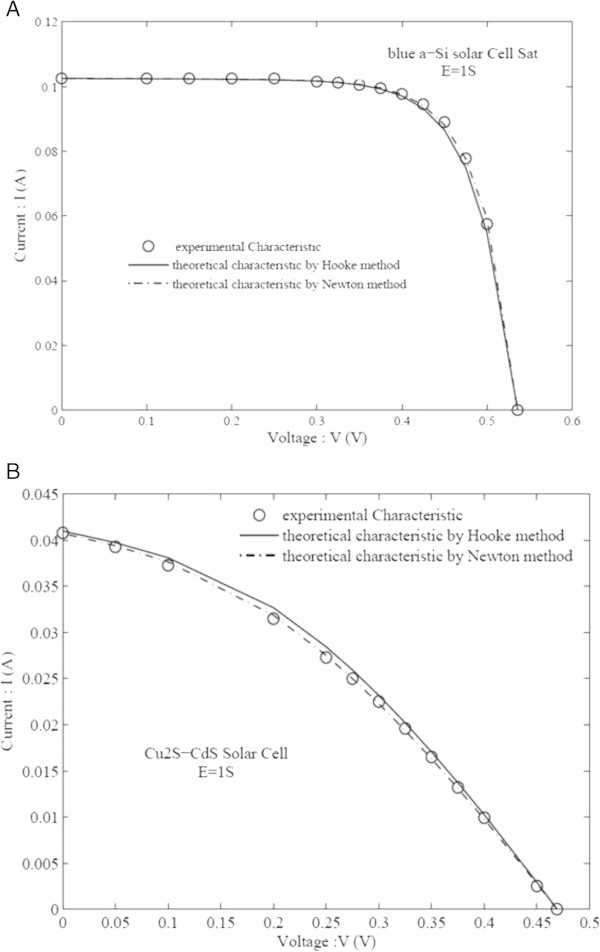
**Experimental I-V Characteristics. (A)** c-Si blue SAT solar cell. **(B)** Frontwall Cu2S-CdS solar cell.

The graphs presented in Figure 4A and B show how close the values calculated by the two used numerical methods to the experimental ones.

**Figure 5:**
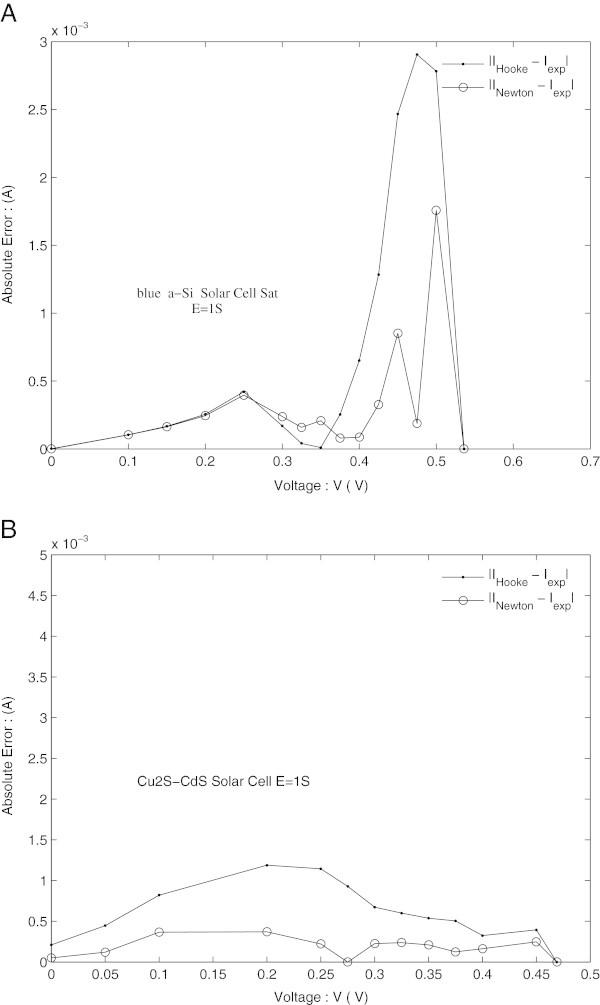
**Absolute error between experimental and calculated current. (A)** c-Si blue SAT solar cell. **(B)** Frontwall Cu2S CdS solar cell.

These figures are very sensitive to the effects of the circuit parameters with localized constants and especially to the quality of the cell. Figure 5A and B outline the absolute errors between the experimental and calculated current as a function of the cell bias voltage by the two numerical methods. Although *D* values of the SAT solar cell in the state of the art are weaker than those of Cu_2_S-CdS solar cell with significant losses; absolute error (Figure 5A) goes to a maximum at *V*_*oc*_-neighborhood. This maximum is weaker in the case of Newton’s method, so denoting a better convergence of this method compared to Hooke’s. Although in the case of the Cu_2_S-CdS solar cell with significant losses this indeterminacy on *R*_*s*_ disappears, the calculated I-V curves show a better convergence of Newton’s method.

## Conclusion

In this study a simple and specific method (without approximations) was proposed to extract intrinsic electrical parameters of the one-diode solar cell model under AM1 illumination (1S).

The proposed approach includes parasite and dissipative elements such as series resistance *R*_*s*_ and shunt resistance *R*_*sh*_.

The use of the Lambert W-function has allowed to express explicitly the current *I* as a function of the voltage *V* from the modified Shockley’s eq. (1).

However, it is important to highlight that the proposed method is valid for all measured I-V characteristics under any illumination intensity.

The implicit functions theorem was used to demonstrate the uniqueness of the solution. The physical considerations of the problem have also been taken into account. This procedure has proved the uniqueness of the solution.

Two different numerical methods: Newton’s method and Hooke-Jeeves’s were used to determine these parameters and reconfirm the uniqueness of the solution.

To prove the authenticity of this extraction method, two different types of solar cell structure were used: a SAT monocrystalline silicon homostructure in the state of the art, and a frontwall Cu_2_S-CdS heterostructure with significant losses.

Moreover, as MATLAB has limitations toward large numbers manipulation (≥ exp (100)), MAPLE software was selected for this calculation.

For the two cell types, both used numerical methods converge in each of cases, towards two series of theoretical results with relative accuracy about 3% in the case of the weak series resistance.

## Nomenclature

*T*: Thermodynamic Temperature in Kelvin (K)

*q*: Electron Charge = 1.602*10^-19^ C

*k*: Boltzmann constant = 1.38*10^-23^ J/K

*V*_*T*_: Thermal voltage = kT/q

*V*_*oc*_: Open circuit voltage

*I*_*sc*_: Short-current voltage

*I*: Output current

*V*: Output voltage

*I*_*max*_: Maximum power current

*V*_*max*_: Maximum power voltage

*P*_*max*_: maximum power

*I*_*ph*_: Photocurrent

*I*_*s*_: Diode reverse saturation current

*I*_*oc*_: Calculated current at the (V_oc_, 0) point

*V*_*sc*_: Calculated voltage at the (0, I_sc_) point

*n*: Diode quality factor

*R*_*sh*_: shunt resistance

*R*_*sh0*_: Differential Resistance at the (0, I_sc_) point

*R*_*s*_: Series resistance

*R*_*s0*_: Differential resistance at the (V_oc_, 0) point

*W*: Lambert’s function

*C*_*+*_: the set of complex numbers with positive real part.
